# A study on the measurement of the reference range of the whole blood viscosity in Thoroughbred horses

**DOI:** 10.17221/24/2024-VETMED

**Published:** 2024-11-22

**Authors:** Yongsu Ha, Kangwoo Yi, Chul Park, Gyumin Kim, Daeyoung Choi, Jumjae Lee, Namsoo Kim

**Affiliations:** Department of Veterinary Surgery, College of Veterinary Medicine, Jeonbuk National University, Republic of Korea

**Keywords:** haemorheology, shear rates, viscometer

## Abstract

This study aimed to measure the whole blood viscosity (WBV) in racehorses using a new viscometer and establish reference values, as well as to investigate the correlation between the WBV and the haematological parameters and serum chemistry. WBV measurements were conducted on 51 Thoroughbred horses using a novel U-shaped scanning capillary-tube viscometer. The reference values for the WBV were determined at various shear rates ranging from 1 s^–1^ to 1 000 s^–1^. Correlation analyses were performed to examine the correlation between the WBV and the haematological and serum chemistry parameters. The findings provide valuable reference data for the WBV in Thoroughbred horses and enhance understanding of the relationship between the WBV and routine blood tests in equine health assessment.

General blood tests, such as haematological and serum chemical tests, provide information to understand the current condition and disease of the patient (Aderemi 2004). Diseases related to vascular disorders are limited to being diagnosed early by routine blood tests such as haematology and serum chemistry. These common blood tests make it difficult to suggest treatment guidelines. Recently, there has been a lot of interest in haemorheology. Haemorheology is the study of analysing the blood flow changed by red blood cell (RBC) aggregation and deformation and interaction with blood vessel walls. Therefore, vascular disorders are very closely related to haemorheology (Cokelet and Meiselman 2007). The main parameter of hemorheology is the whole blood viscosity (WBV) which depends on the shear stresses and pressure gradients. WBV is determined by the haematocrit, plasma protein concentration, plasma viscosity, erythrocyte aggregation, deformability, and mechanical properties of red blood cells (RBCs) (Baskurt and Meiselman 2003). WBV is a function of the quantity and quality of suspended materials, which are RBCs and diverse plasma proteins (Hershko and Carmeli 2003). Thus, a study on the correlation between the WBV and haematology, and serum chemistry is essential for the better understanding of haemorheology. In human medicine, many studies on WBV have already been researched. For instance, the association with WBV and coronary disease, blood pressure, ageing, etc. have been revealed (Nicolaides et al. 1977; Letcher et al. 1981). However, although blood viscosity has been studied, only some limited research on blood viscosity in horses exists (Andrews et al. 1992; Fedde and Erickson 1998). Therefore, the deeper understanding of normal reference values of blood viscosity is required for the early diagnosis of conditions such as circulatory problems, cardiac insufficiency, retinal haemorrhage, kidney disease and hypertension (Williams and Goldschmidt 1982; Rosenson et al. 1996; Ramaiah et al. 2002).

In racing horses, the haematological and flow characteristics, such as the WBV, can be used as parameters for recovery to resting values (Wood and Fedde 1997). Thus, it could be important to study the WBV in racing horses for anticipating the racing ability.

Viscometers, which are customarily used today, are capillary viscometers, falling-body viscometers and rotational viscometers. However, there are some limitations, most of which are due to the measurement of certain viscosities, the constant shear rates and the mechanical devices themselves, often making experimental errors. Moreover, conventional viscometers are hard to handle. In this study, a new type of capillary tube viscometer, a U-shaped scanning capillary-tube viscometer [U-SCTV (BVD-PRO1^®^; Bio-visco, Inc., Jeonju-Si, Republic of Korea)], is used. This viscometer can continuously measure the yield stress and viscosity of the whole blood over a wide range of shear rates from 1 s^–1^ to 1 000 s^–1^ (Kim et al. 2000).

The purpose of this study is to measure the WBV using a new viscometer and then establish reference values and confirm the correlation between the WBV and the haematology, serum chemistry in Thoroughbred horses.

## MATERIAL AND METHODS

### Experimental animals

Fifty-one healthy, adult Thoroughbred horses (34 stallions, 17 mares) were used following Institutional Animal Care and Use Committee (IACUC) review. The horses weighed between 445.8–531.4 kg (median 498.7 kg), and the ages ranged from 7 to 18 years (median 14 years). Before being included in the study, all the horses were reared under uniform conditions, receiving identical feed and exercise management protocols. A thorough physical examination conducted by a licensed veterinarian ensured the absence of any current gastrointestinal or other severe systemic diseases. Also, there was no history of drug administration in the week preceding the study.

Blood samples (10 ml) were obtained from the jugular vein. The blood was sampled during the day without exercise. Each withdrawn blood sample was divided into Ethylenediaminetetraacetic acid (EDTA)-coated tubes (3 ml each) and plain tubes (4 ml). A constant volume (3 ml) of an EDTA-anticoagulated blood sample was used for the complete blood count (CBC) and WBV measurements. The plain tube sample was used for the serum chemistry. The blood sample was kept at a uniform temperature (37.0 °C). The CBC and serum chemistry were measured within 4 h after the blood collection. The blood samples for the serum chemistry were centrifuged at 1 800 *g*, 4 °C for 10 minutes. The supernatant was separated and measured within 4 hours.

### Haematological analysis

The CBC was measured using an automatic blood cell counter (Vet ABC^®^; ABX Diagnostics, Montpellier, France). The red blood cell count (RBC, 10^6^/mm^3^), haemoglobin concentration (HGB, g/l), haematocrit (HCT, %), white blood cell count (WBC, 10^3^/mm^3^), platelet (PLT, 10^3^/mm^3^), mean corpuscular volume (MCV, μm^3^), mean corpuscular haemoglobin (MCH, pg), mean cell haemoglobin concentration (MCHC, g/l), red cell distribution (RDW, %), mean platelet volume (MPV, μm^3^) were measured.

### Serum chemical analysis

The serum chemistry was measured using an automatic chemistry analyser (Hitachi Auto Analyser 7020^®^; Hitachi Co., Tokyo, Japan). The concentration of albumin (ALB, g/l), alkaline phosphatase (ALP, nkat/l), alanine transaminase (ALT, nakt/l), amylase (AMY, nakt/l), total bilirubin (TBIL, mg/l) blood urea nitrogen (BUN, mg/l), calcium (Ca, mg/l), cholesterol (CHOL, mg/l), creatinine (CRE, mg/l), glucose (GLU, mg/l), phosphorus (P, mg/l), total protein (TP, g/l), globulin (GLOB, g/l), sodium (Na^+^, mmol/l), potassium (K^+^, mmol/l), chloride (Cl^–^, mmol/l) were measured.

### Whole blood viscosity analysis

The WBV (cP) measurements were made by a U-shaped scanning capillary-tube viscometer (BVD-PRO1^®^; Bio-visco, Inc., Jeonju-Si, Republic of Korea) which is capable of measuring the yield stress and viscosity of whole blood continuously over a whole range of shear rates from 1 s^–1^ to 1 000 s^–1^, which calculates the viscosity using a Casson fluid model.

### Statistical analyses

In this study, for analysing WBV data, the mean, standard deviation (SD) and 95% confidence intervals were calculated. Pearson’s correlation coefficient analysis was used to research the correlation between the WBV and the haematology and serum chemistry.

After the classification of horses into three distinct age groups (7 years or younger, older than 7 years but up to 14 years, and those older than 14 years) a one-way analysis of variance (ANOVA) was performed in order to evaluate and assess potential differences in the WBV among these age groups. SPSS v22.0 (PASW Statistics; IBM Co., Armonk, NY, USA) was used for the statistical analysis. *P*-values under 0.05 or less were considered statistically significant.

## RESULTS

### Haematological analysis

The haematological values were measured using an automatic blood cell counter and results were presented in Table 1 (Vet ABC^®^; ABX Diagnostics, Montpellier, France).

**Table 1:** Haematological values in the blood from the Thoroughbred horses (*n* = 51)

Haematological parameter	Mean ± SD	Reference values
HCT	38.15 ± 5.61	32–46
MCV	46.57 ± 6.59	37–55
MCH	15.99 ± 2.32	13–19
MCHC	343.5 ± 5.8	310–350
HGB	131.1 ± 20.0	110–190
WBC	5.57 ± 1.12	5.4–14
PLT	138.75 ± 29.87	100–350
RBC	8.11 ± 1.43	6.8–12.9

Reference values from Axon and Palmer (2008)

HCT = haematocrit; HGB = haemoglobin; MCH = mean corpuscular haemoglobin; MCHC = mean cell haemoglobin concentration; MCV = mean corpuscular volume; PLT = platelet; RBC = red blood cell; SD = standard deviation; WBC = white blood cell

### Serum chemical analysis

The serum chemistry was measured using an automatic chemistry analyser and results were presented in Table 2 (Hitachi Auto Analyzer 7020^®^; Hitachi Co., Tokyo, Japan).

**Table 2:** Biochemistry values in the blood from the Thoroughbred horses (*n* = 51)

Haematological parameter	Mean ± SD	Reference values
ALP	1 774.19 ± 493.27	1 817–5 251
TP	60.31 ± 4.40	46–69
CREA	13.9 ± 2.7	6–18
CHOL	722.4 ± 123.2	510–1 090
P	22.1 ± 6.7	19–54
ALB	33.5 ± 4.9	25–42
BUN	133.9 ± 26.1	80–270

Data are expressed as the median; Reference values from Charles River Baseline Haematology (Charles River Laboratories 1982)

ALB = albumin; ALP = alkaline phosphatase; BUN = blood urea nitrogen; CHOL = cholesterol; CREA = creatinine; P = phosphorus; SD = standard deviation; TP = total protein

### Reference values of the WBV

The Mean, SD and 95% confidence intervals were acquired by statistical analysis. Owing to the number of horses (51 thoroughbred horses), 95% confidence intervals were used as the reference range. The reference values of the WBV in horses are presented in Table 3.

**Table 3:** Reference values of whole blood viscocity for the Thoroughbreds (*n* = 51)

Shear rates (SR)		Mean ± SD	95% confidence intervals	
			lower	upper
Whole blood viscosity (cP)	SR 1 s^–1^	31.72 ± 12.46	28.21	35.22
	SR 5 s^–1^	13.09 ± 4.65	11.79	14.40
	SR 10 s^–1^	9.87 ± 3.36	8.93	10.82
	SR 50 s^–1^	6.29 ± 2.00	5.73	6.85
	SR 100 s^–1^	5.49 ± 1.58	5.05	5.93
	SR 150 s^–1^	5.15 ± 1.44	4.75	5.56
	SR 300 s^–1^	4.69 ± 1.33	4.32	5.07
	SR 1 000 s^–1^	4.15 ± 1.31	3.78	4.52

**Figure 1:**
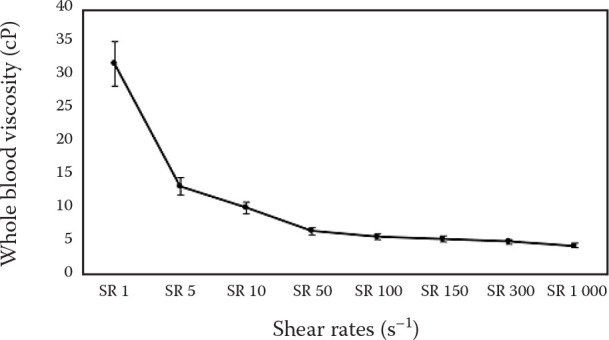
Mean WBV (cP) at shear rates from 1 s^–1^ to 1 000 s^–1^ in the Thoroughbred horses The graph illustrates the mean values with 95% confidence intervals SR = shear rates; WBV = whole blood biscosity

The graph of the mean WBV (cP) at shear rates from 1 s^–1^ to 1 000 s^–1^ in the Thoroughbred horses is illustrated in Figure 1.

### Correlation analysis

**Figure 2:**
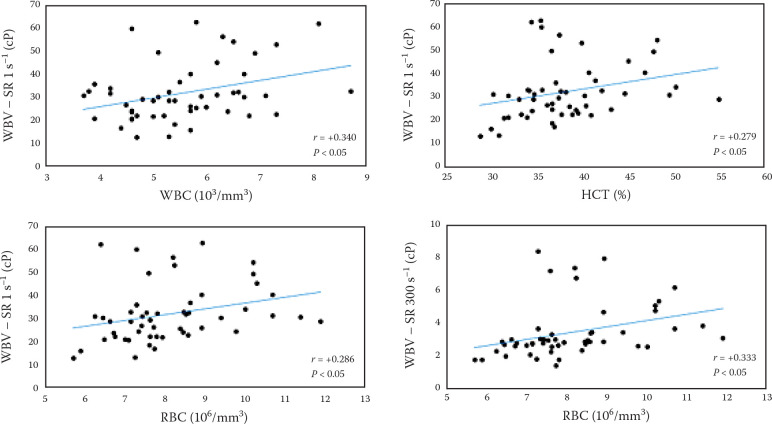
Scatter diagram with *r* values between the diastolic (shear rate 1 s^–1^) and systolic (shear rate 300 s^–1^) blood viscosity and the WBC, RBC and HCT HCT = haematocrit; RBC = red blood cell; SR = shear rates; WBC = white blood cell; WBV = whole blood viscosity

**Figure 3:**
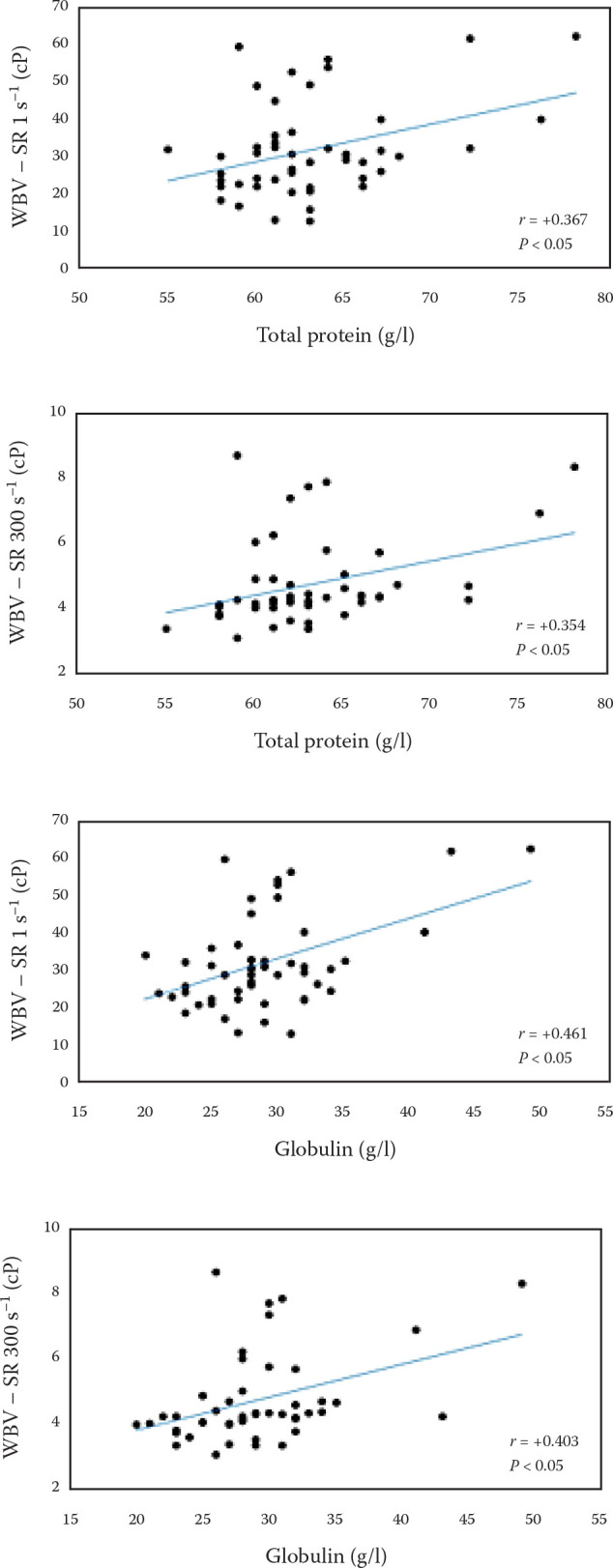
Scatter diagram with *r* values between the diastolic (shear rate 1 s^–1^) and systolic (shear rate 300 s^–1^) blood viscosity and the TP and GLOB GLOB = globulin; SR = shear rates; TP = total protein; WBV = whole blood viscosity

**Figure 4:**
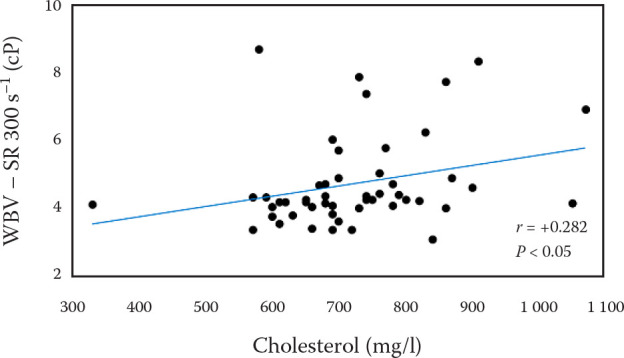
Scatter diagram with *r* values between the systolic (shear rate 300 s^–1^) blood viscosity and the CHOL CHOL = cholesterol; SR = shear rates; WBV = whole blood viscosity

The coefficient of correlation, the *r* values resulting from the correlation analysis, will be examined in more detail later in Tables 5 and 6. The *r* values are presented where there is a significant correlation (*P* < 0.05 or less). Scatter diagrams with the *r* correlation values are illustrated in Figures 2–4.

### Correlation between the WBV and the haematology

The RBC was statistically correlated with the WBV over the whole range of the shear rates (Table 4). The WBC, HGB and HCT were statistically correlated with the WBV over a low range of shear rates (Table 4). The correlation between the WBC, RBC, HGB, HCT, and WBV was illustrated by a scatter diagram with r values (Figure 2).

**Table 4:** Correlation between the WBV and the haematology

Shear rates		WBC	RBC	HGB	HCT
Whole blood viscosity (cP)	SR 1 s^–1^	0.340*	0.286*	–	0.279*
	SR 5 s^–1^	0.346*	0.299*	0.285*	0.296*
	SR 10 s^–1^	0.346*	0.303*	0.291*	0.301*
	SR 50 s^–1^	0.342*	0.306*	0.298*	0.307*
	SR 100 s^–1^	0.297*	0.327*	0.291*	0.300*
	SR 150 s^–1^	–	0.330*	0.280*	0.288*
	SR 300 s^–1^	–	0.333*	–	–
	SR 1 000 s^–1^	–	0.326*	–	–

*P < 0.05

HCT = haematocrit; HGB = haemoglobin; RBC = red blood cell; SR = shear rates; WBC = white blood cell; WBV = whole blood viscosity

### Correlation between the WBV and the serum chemistry

The TP and GLOB concentration statistically correlated with the WBV over the whole range of shear rates (Table 5). The Cl concentration correlated with the low shear rates (Table 5). The CHOL concentration correlated with the high shear rates (Table 5). The correlation between TP, GLOB, CHOL, Cl and WBV was illustrated by the scatter diagrams with *r* values (Figures 3 and 4).

**Table 5:** Correlation between the WBV and the serum chemistry

Shear rates		TP	GLOB	Cl	CHOL
Whole blood viscosity (cP)	SR 1 s^–1^	0.367**	0.461**	–	–
	SR 5 s^–1^	0.351*	0.441**	0.281*	–
	SR 10 s^–1^	0.341*	0.428**	0.286*	–
	SR 50 s^–1^	0.317*	0.397**	0.292*	–
	SR 100 s^–1^	0.333*	0.402**	–	–
	SR 150 s^–1^	0.338*	0.401**	–	–
	SR 300 s^–1^	0.354*	0.403**	–	0.282*
	SR 1 000 s^–1^	0.385**	0.411**	–	0.310*

*P < 0.05, **P < 0.01

Cl = chloride; CHOL = cholesterol; GLOB = globulin; SR = shear rates; TP = total protein; WBV = whole blood vicosity

### Difference in the WBV according to the age groups

The difference in the WBV according to the age groups after dividing the three groups was statistically significant at a shear rate 1 000 s^–1^. As a result of Scheffe’s test, the “Over 14 years old” group’s WBV is higher than the other groups at the shear rate 1 000 s^–1^ (Table 6).

**Table 6:** Difference in the WBV according to the age groups

Shear rates		Under 7 years (*N* = 25)	7 years to 14 years (*N* = 19)	Over 14 years (*N* = 7)	*P*-value	Scheffe
Whole blood viscosity (cP)	SR 1 s^–1^	31.69 ± 11.92	30.23 ± 11.52	35.85 ± 17.36	0.604	–
	SR 5 s^–1^	13.13 ± 4.39	12.42 ± 4.34	14.78 ± 6.44	0.527	–
	SR 10 s^–1^	9.92 ± 3.16	9.35 ± 3.17	11.14 ± 4.64	0.493	–
	SR 50 s^–1^	6.34 ± 1.84	5.93 ± 1.93	7.09 ± 2.69	0.426	–
	SR 100 s^–1^	5.54 ± 1.44	5.12 ± 1.43	6.33 ± 2.28	0.223	–
	SR 150 s^–1^	5.20 ± 1.30	4.78 ± 1.25	6.01 ± 2.13	0.151	–
	SR 300 s^–1^	4.72 ± 1.21	4.32 ± 1.09	5.60 ± 1.98	0.091	–
	SR 1 000 s^–1^	4.16 ± 1.21	3.75 ± 1.06	5.16 ± 1.86	0.048*	b < c

*P < 0.05

SR = shear rates; WBV = whole blood viscosity

## DISCUSSION

To our knowledge, this is the first study of whole blood viscosity values measured concurrently from a population of healthy horses that were raised in the same environment with consistent feeding and exercising management. The erythrocyte sedimentation rate (ESR) of horses is more rapid than other animals, the WBV was measured immediately at a stable condition after blood collection (Windberger et al. 2003). One of other main factors affecting the WBV is exercise in racing horses (Wood and Fedde 1997). So, the blood collection was conducted on the same day of the week, which was a rest day. The horses were under the same exercise regimes each week. The general health condition is also one of the main factors affecting the WBV (Baskurt and Meiselman 2003). In this experiment, the HCT, MCV, MCH, MCHC and TP of all the horses were measured. The means of the HCT, MCV, MCH, MCHC and TP were well within the reference values (Table 3). This study was controlled for various factors, such as rapid ESR errors, the effects of exercise and overall health to reduce any potential errors associated with these variables. So, we obtained more stable standard WBV values in the Thoroughbred horses.

In a previous study, the WBVs at 0.7 s^–1^, 2.4 s^–1^, and 94 s^–1^ were 38.173, 20.179, and 5.175 cp with 40% HCT, respectively, in horses consisting of two breeds (Windberger et al. 2003). Due to the HCT concentration differences in each study, an absolute comparison between the previous result and the present result is not available. Moreover, the previous study was conducted only at specific shear rates and that data cannot fully reflect the shear-thinning haemorheological characteristics of whole blood (Kim et al. 2000). However, the data from this study were measured over a rage of shear rates continuously from 1 s^–1^ to 1 000 s^–1^. So, the mean and reference range in this study were suggested over the whole range of the shear rates from 1 s^–1^ to 1 000 s^–1^ (Koh et al. 2014).

As the shear forces increase, the blood viscosity decreases. For this reason, blood is described as a non-Newtonian fluid (Cokelet 2011). Two RBC characteristics, deformability and aggregability, affect the viscosity of blood at high and low shear rates. In high shear rates, the deformability of RBCs is a major factor that affects the WBV, on the other hand, the aggregability of RBCs is a significant factor to determine the WBV in low shear rates. Under low shear conditions, the non-Newtonian properties of blood become deeper through the rouleaux formation of the RBCs, which means that the RBCs coagulate into coin piles. Such coin-like agglomeration tends to increase the frictional resistance. This increases the blood viscosity at low shear rates (Baskurt and Meiselman 2003). Under high shear conditions, RBCs, which are flexible cells, can be more easily converted into ellipsoidal structures. This transition promotes the orientation of the flow through the blood stream and reduces the blood viscosity at high shear rates (Chien 1987; Baskurt and Meiselman 2003). For all those reasons, blood viscosity is primarily affected by the RBCs. The correlation between the WBV and the RBC, HGB, HCT has been shown in many previous studies (Hershko and Carmeli 1970; Johnn et al. 1992; Popel et al. 1994; Bodey and Rampling 1998; Baskurt and Meiselman 2003). Similar to the previous study, the RBC, HGB, and HCT are statistically correlated with the WBV in this study. The RBCs are especially statistically correlated with the WBV over the whole range of shear rates (*P* < 0.05).

The correlation between the TP, GLOB, CHOL concentration, and WBV can be found in previous reports. The erythrocyte aggregation correlated with the total plasma protein concentration. The plasma viscosity correlated with the total serum protein (Rosenson et al. 1996; Windberger et al. 2003). A correlation between the TP, GLOB and CHOL concentration and the WBV was observed in this study, but not in previous studies. The TP was statistically correlated with the WBV over the whole range of shear rates. At the diastolic shear rate (1 s^–1^), the *r* value between the TP and WBV was 0.367 (*P* < 0.01). At the systolic shear rate (300 s^–1^), the *r* value between the TP and WBV was 0.385 (*P* < 0.05).

Immunoglobulin affects the WBV by inducing RBC aggregation (Kwaan 2010). The degree of hyperviscosity correlates with the size of the paraprotein and the severity of the hyperglobulinemia. Large molecules are more effective in increasing the serum viscosity. Therefore, hyperviscosity is shown commonly in macroglobulinemia. Furthermore, IgA aggregates molecules to produce polymers that have a large effect on the serum viscosity (Williams and Goldschmidt 1982; Ramaiah et al. 2002; Zaldivar-Lopez et al. 2011). In this study, GLOB was statistically correlated with the WBV over the whole range of shear rates (*P* < 0.01). At the diastolic shear rate (1 s^–1^), the *r* value between the GLOB and WBV was 0.461. At the systolic shear rate (300 s^–1^), the *r* value between the TP and WBV was 0.403.

In previous studies, it was shown that high-density lipoprotein cholesterol (HDL) has a specific viscoelastic property (Epand et al. 1994). HDL reduces the RBC aggregation and allows the RBCs to maintain deformability (Stamos and Rosenson 1995). In this study, the correlation between the CHOL and WBV was statistically significant at high shear rates (*P* < 0.05). At the systolic shear rate (300 s^–1^), the *r* value between CHOL and WBV was 0.282. As described earlier, cholesterol maintains the RBC deformability and reduces the RBC aggregation, so the higher the shear rate, the higher the correlation coefficient.

Cl is a major anion and plays a leading role in acid-base equilibrium and osmotic pressure. Changes in the water balance cause changes in the chloride and sodium ion concentrations (Morais 1992). As blood is dehydrated, the blood viscosity increases. Changes in water, such as blood dehydration, affect the Cl, sodium concentration, and blood viscosity. Therefore, we think there is a causal relationship. In this study, a correlation between the Cl and WBV was presented at low shear rates (*P* < 0.05).

Several studies have shown that impaired blood fluidity in advanced age affects the blood viscosity (Simmonds et al. 2013). In this study, the WBV value was statistically significant according to the ages at specific shear rates (*P* < 0.05). As a result of Scheffe’s post-test, the WBV value at the shear rate 1 000 s^–1^ in the over 14-year-old group is higher than the 7–14-year-old group.

This study has some limitations. The control of hormonal factors was not achieved and due to an insufficient sample volume, the fibrinogen was not measured. Therefore, further studies related to the whole blood viscosity would be need to be conducted to confirm the correlation between those factors.

In conclusion it can be stated, that the average and reference values of the whole blood viscosity were investigated in Thoroughbred horses. At the diastolic shear rate (1 s^–1^), the reference values of WBV were from 28.21 to 35.22. At the systolic shear rate (300 s^–1^), the reference values of WBV were from 4.32 to 5.07. This study suggested a correlation between the WBV and regular blood tests, such as the haematology and serum chemistry in Thoroughbred horses.
